# Supplementary material for: Drug repurposing for aging research using model organisms
Source: Aging Cell. 2017 Jun 16;16(5):1006–15. doi: 10.1111/acel.12626 (PMC5595691; doi:10.1111/acel.12626)
Supplement: Supplementary file 7 — Data S1 Zip‐Archive of all report cards. [file ACEL-16-1006-s007.zip › RC_XFE.pdf]

# XFE

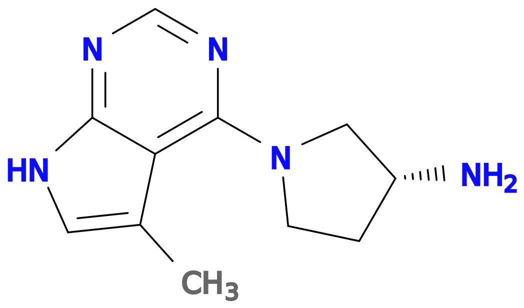


**Database identifiers**

| | |
|---|---|
| ChEMBLCompound | CHEMBL1173273 |

## Ranking

| | Rank | Score |
|---|---|---|
| Drosophila | 313/697 | 0.512 |
| C. elegans | 333/591 | 0.177 |

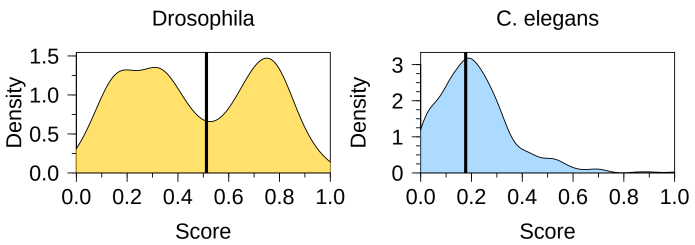


| | Ageing implication | Domain conservation | Binding site conservation | Binding affinity | Bioavailability | Lipinski | Promiscuity | Purchasability | Drug approval | **Total** |
|---|---|---|---|---|---|---|---|---|---|---|
| Drosophila | 1.0 | 0.869 | 0.819 | 0.801 | (0.9) | 0.0 | -0.0 | 0.0 | 0.0 | 0.512 |
| C. elegans | 1.0 | 0.926 | 0.986 | 0.801 | 0.243 | 0.0 | -0.0 | 0.0 | 0.0 | 0.177 |

## Names

No synonyms found

## Roles

ChEBI entry None has no roles

## Status

| | |
|---|---|
| Approved drug (according to ChEMBL) | No |
| Number of Rule of 5 violations | 0 |
| Binding affinity to original target in log units (RF-Score prediction) | 6.39 |
| Burns *C. elegans* bioavailability prediction | -2.46 |

# Compound Target Characteristics

## RAC-alpha serine/threonine-protein kinase

Best gene implication in ageing for this target family came from gene Q94533 annotated in UniProt release 2014_02. Annotation GO 8340 (determination of adult lifespan) was Inferred from Mutant Phenotype

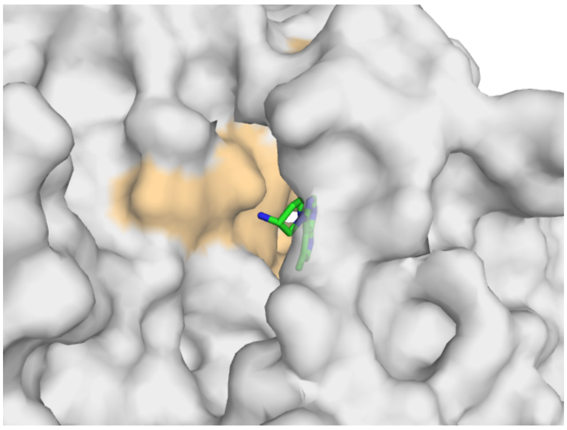

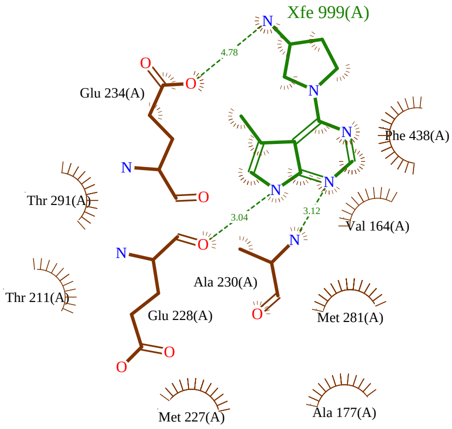


| protein | amino acids contacts (binding site) | | | | | | | | | | |
|---|---|---|---|---|---|---|---|---|---|---|---|
| PDB:3mv5:chainA:P31749 | V | A | T | M | E | A | E | E | M | T | F |
| tr:B3KP61:B3KP61_HUMAN | V | A | T | M | E | A | E | E | M | T | F |
| tr:B7Z5R1:B7Z5R1_HUMAN | V | A | T | M | E | A | E | E | M | T | F |
| tr:B4DG79:B4DG79_HUMAN | V | A | T | M | E | A | E | E | M | T | F |
| tr:M0R0P9:M0R0P9_HUMAN | V | A | T | M | E | A | E | E | M | T | F |
| sp:P31749:AKT1_HUMAN | V | A | T | M | E | A | E | E | M | T | F |
| tr:B0LPE5:B0LPE5_HUMAN | V | A | T | M | E | A | E | E | M | T | F |
| sp:P31751:AKT2_HUMAN | V | A | T | M | E | A | E | E | M | T | F |
| sp:P47196:AKT1_RAT | V | A | T | M | E | A | E | E | M | T | F |
| tr:Q3HSE5:Q3HSE5_RAT | V | A | T | M | E | A | E | E | M | T | F |
| tr:Q8CE74:Q8CE74_MOUSE | V | A | T | M | E | A | E | E | M | T | F |
| sp:Q60823:AKT2_MOUSE | V | A | T | M | E | A | E | E | M | T | F |
| tr:Q3TY95:Q3TY95_MOUSE | V | A | T | M | E | A | E | E | M | T | F |
| sp:P31750:AKT1_MOUSE | V | A | T | M | E | A | E | E | M | T | F |
| tr:H1ZYE3:H1ZYE3_DROME | V | A | V | L | E | L | E | E | L | T | F |
| tr:Q94533:Q94533_DROME | V | A | V | L | E | L | E | E | L | T | F |
| tr:P91656:P91656_DROME | V | A | V | L | E | L | E | E | L | T | F |
| sp:Q9XTG7:AKT2_CAEEL | V | A | T | M | E | A | E | E | L | T | F |
| sp:P11792:SCH9_YEAST | V | A | V | T | D | M | E | E | L | C | F |

| protein | whole protein ident | simil | domain-based ident | simil | contact-based ident | simil |
|---|---|---|---|---|---|---|
| PDB:3mv5:chainA:P31749 | 0.99 | 1.0 | 0.99 | 1.0 | 1.0 | 1.0 |
| tr:B3KP61:B3KP61_HUMAN | 0.64 | 0.74 | 0.86 | 0.96 | 1.0 | 1.0 |
| tr:B7Z5R1:B7Z5R1_HUMAN | 0.87 | 0.87 | 1.0 | 1.0 | 1.0 | 1.0 |
| tr:B4DG79:B4DG79_HUMAN | 0.71 | 0.82 | 0.86 | 0.96 | 1.0 | 1.0 |
| tr:M0R0P9:M0R0P9_HUMAN | 0.65 | 0.76 | 0.79 | 0.9 | 1.0 | 1.0 |
| sp:P31749:AKT1_HUMAN | 1.0 | 1.0 | 1.0 | 1.0 | 1.0 | 1.0 |
| tr:B0LPE5:B0LPE5_HUMAN | 1.0 | 1.0 | 1.0 | 1.0 | 1.0 | 1.0 |
| sp:P31751:AKT2_HUMAN | 0.81 | 0.94 | 0.86 | 0.96 | 1.0 | 1.0 |
| sp:P47196:AKT1_RAT | 0.98 | 0.99 | 0.99 | 1.0 | 1.0 | 1.0 |
| tr:Q3HSE5:Q3HSE5_RAT | 0.81 | 0.94 | 0.86 | 0.96 | 1.0 | 1.0 |
| tr:Q8CE74:Q8CE74_MOUSE | 0.62 | 0.7 | 0.86 | 0.96 | 1.0 | 1.0 |
| sp:Q60823:AKT2_MOUSE | 0.81 | 0.94 | 0.86 | 0.96 | 1.0 | 1.0 |
| tr:Q3TY95:Q3TY95_MOUSE | 0.81 | 0.94 | 0.86 | 0.96 | 1.0 | 1.0 |
| sp:P31750:AKT1_MOUSE | 0.98 | 0.99 | 0.99 | 1.0 | 1.0 | 1.0 |
| tr:H1ZYE3:H1ZYE3_DROME | 0.3 | 0.57 | 0.42 | 0.78 | 0.64 | 0.82 |
| tr:Q94533:Q94533_DROME | 0.26 | 0.5 | 0.43 | 0.79 | 0.64 | 0.82 |
| tr:P91656:P91656_DROME | 0.21 | 0.4 | 0.43 | 0.79 | 0.64 | 0.82 |
| sp:Q9XTG7:AKT2_CAEEL | 0.48 | 0.75 | 0.59 | 0.85 | 0.91 | 0.99 |
| sp:P11792:SCH9_YEAST | 0.19 | 0.42 | 0.45 | 0.8 | 0.45 | 0.6 |

**S6k (FBgn0015806) associated phenotypes**

cell autonomous, chemical resistant, decreased cell number, decreased cell size, feeding behavior defective, heat sensitive, increased cell growth, increased cell number, increased cell size, large body, long lived, memory defective, neuroanatomy defective, neurophysiology defective, nutrition conditional, partially, partially lethal - majority die, short lived, size defective, small body, somatic clone

(Information from FlyBase)

**akt-2 (WBGene00000103) associated phenotypes**

fat content increased, germ cell hypersensitive ionizing radiation, hypersensitivity to mutagen

(Information from WormBase)

**akt-2 (UniProt:Q9XTG7) annotation**

**Function**: Acts downstream of age-1 and pdk-1 in the daf-2/insulin receptor-like transduction pathway. Essential role in regulating developmental arrest at the dauer stage. Phosphorylates Forkhead-related daf-16 and the longevity-promoting skn-1 transcription factors, which inhibits their entry into the nucleus and antagonizes their functions. Role in immune function and pathogen resistance. (PubMed:10364160, PubMed:11381260, PubMed:11747825, PubMed:15068796, PubMed:18358814, PubMed:18782349, PubMed:9716402).

**Cofactor**:Mg(2+)

**Enzyme regulation**: Phosphorylated and activated by pdk-1. (PubMed:15068796).

**Subunit**: Interacts with pdk-1, sgk-1, akt-1 and daf-16. Part of a complex containing sgk-1, akt-1 and akt-2. (PubMed:15068796).

**Tissue specificity**: Expressed in neurons, muscle cells of the pharynx, rectal gland cells, and spermatheca. (PubMed:9716402).

**Developmental stage**: Expressed in late stage embryos and throughout life. (PubMed:9716402).

**Disruption phenotype**: Defective egg-laying and increased resistance to pathogens. Simultaneous knockdown of akt-1 and akt-2 result in dauer formation and a weak extension to life span. (PubMed:15068796, PubMed:18782349).

(Information from UniProt)
